# Supplementary material for: Siderophore and indolic acid production by Paenibacillus triticisoli BJ-18 and their plant growth-promoting and antimicrobe abilities
Source: PeerJ. 2020 Jul 14;8:e9403. doi: 10.7717/peerj.9403 (PMC7367057; doi:10.7717/peerj.9403)
Supplement: Supplemental Information 1 [file peerj-08-9403-s001.docx]

## Cytotoxicity bioassay

An analysis of the cytotoxic bioactivity on tumor cells was performed following the MTT method for compounds **1**-**6** (Carmichael et al., 1987). The tumor cell lines consisted of the leukemia cell (K562), pancreatic cancer cell (AsPC-1), human colon cancer cell (SW480), and the human hepatoma carcinoma cell (HepG2). A549 and ASPC-1 were inoculated in Dulbecco's modified eagle medium (DMEM, Thermo Fisher Scientific Inc.), while K562 was fermented in Roswell Park Memorial Institute 1640 medium (Thermo Fisher Scientific Inc.). The tumor cells were incubated to a density of 10^4^ cells/100μL, and 100μL of suspension solution was transferred to each well in a 96-well clear plate. Cells attached at 37°C in a moist atmosphere of 5% CO_2_ for 1 day and then the medium of each well was replaced by an isovolumetric corresponding medium mixed with gradient concentrations of the isolated samples for 2 days. Cisplatin (Sigma-Aldrich Co., purity ≥ 99%) and taxol (J&K Scientific Ltd., purity ≥ 98%) were chosen as positive controls. 5 μL of 5 mg/mL tetrazolium MTT was mixed with the solution in each well. The supernatant was discarded after a 4 hour incubation period and 150 μL of DMSO was mixed in each well. The SpectraMax 190 microplate reader (Molecular Devices Inc.) was employed to detect OD_540_. The inhibitory activity values were determined as follows: cytotoxic rate (%) = (100-100×OD_treated_/OD_control_)% and obtained as mean ±SD of three parallel experiments. The result showed that deshydroxylferritriacetylfusigen had weak cytotoxicity against AsPC-1 with IC_50_ value of 81.2±3.9 μM, while cis-platinum has an IC_50_ value of 5.3±0.9 μM.
