# Supplementary figures and images for: Siderophore and indolic acid production by Paenibacillus triticisoli BJ-18 and their plant growth-promoting and antimicrobe abilities
Source: PeerJ. 2020 Jul 14;8:e9403. doi: 10.7717/peerj.9403 (PMC7367057; doi:10.7717/peerj.9403)

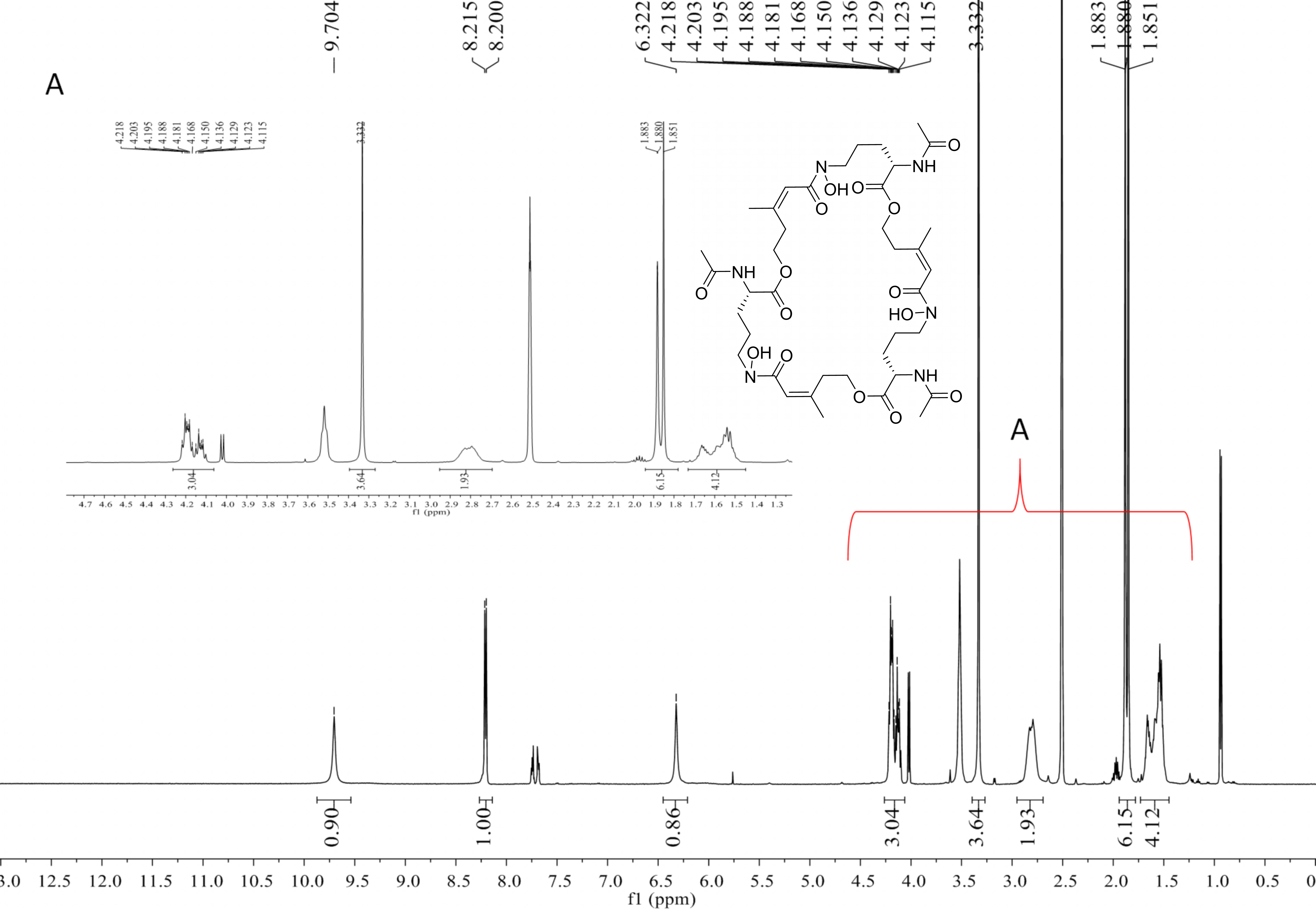

Supplement: Supplemental Information 2 — DMSO-d _6, 500 MHz. [file peerj-08-9403-s002.png]

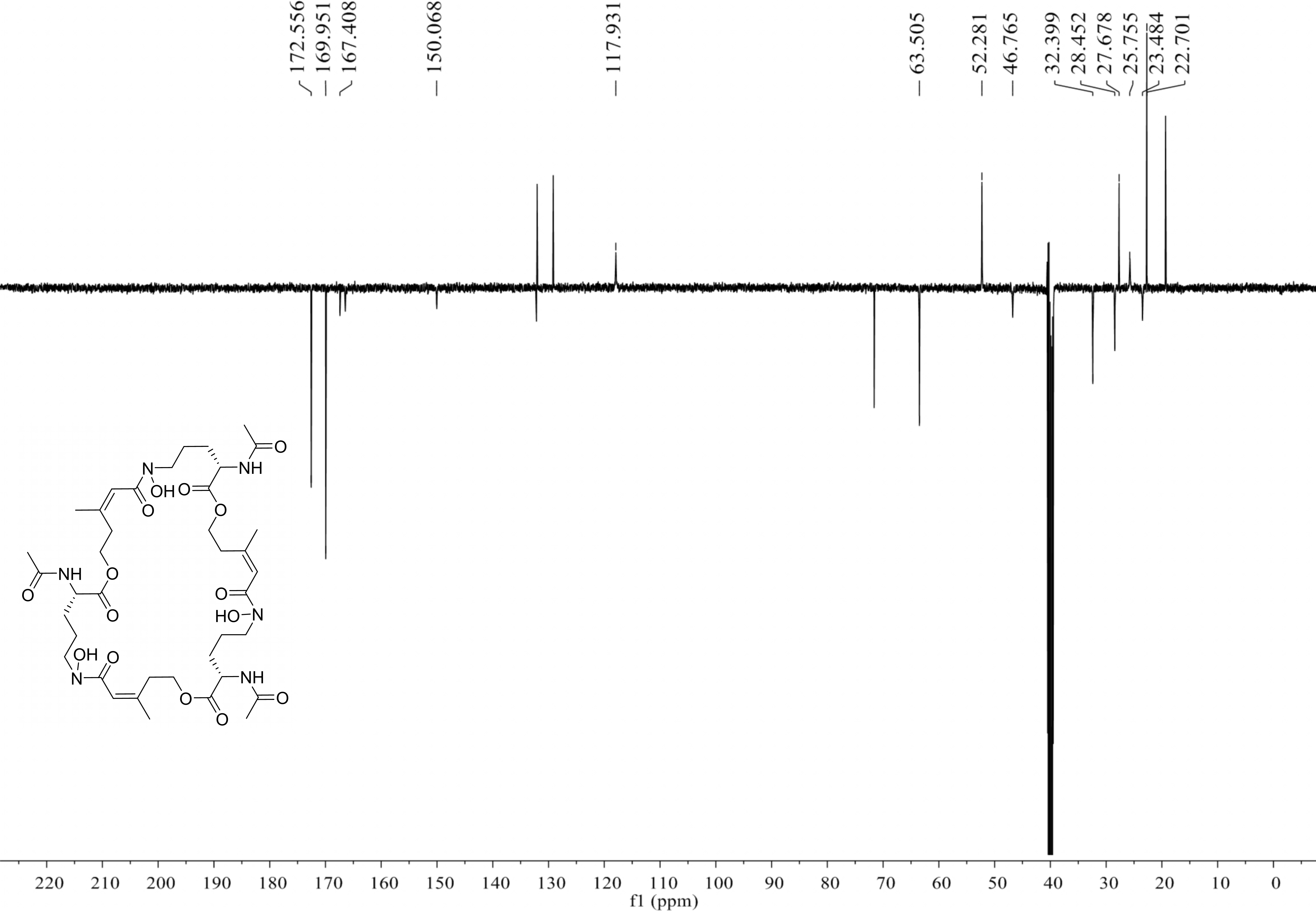

Supplement: Supplemental Information 3 — DMSO-d 6, 125 MHz. [file peerj-08-9403-s003.png]

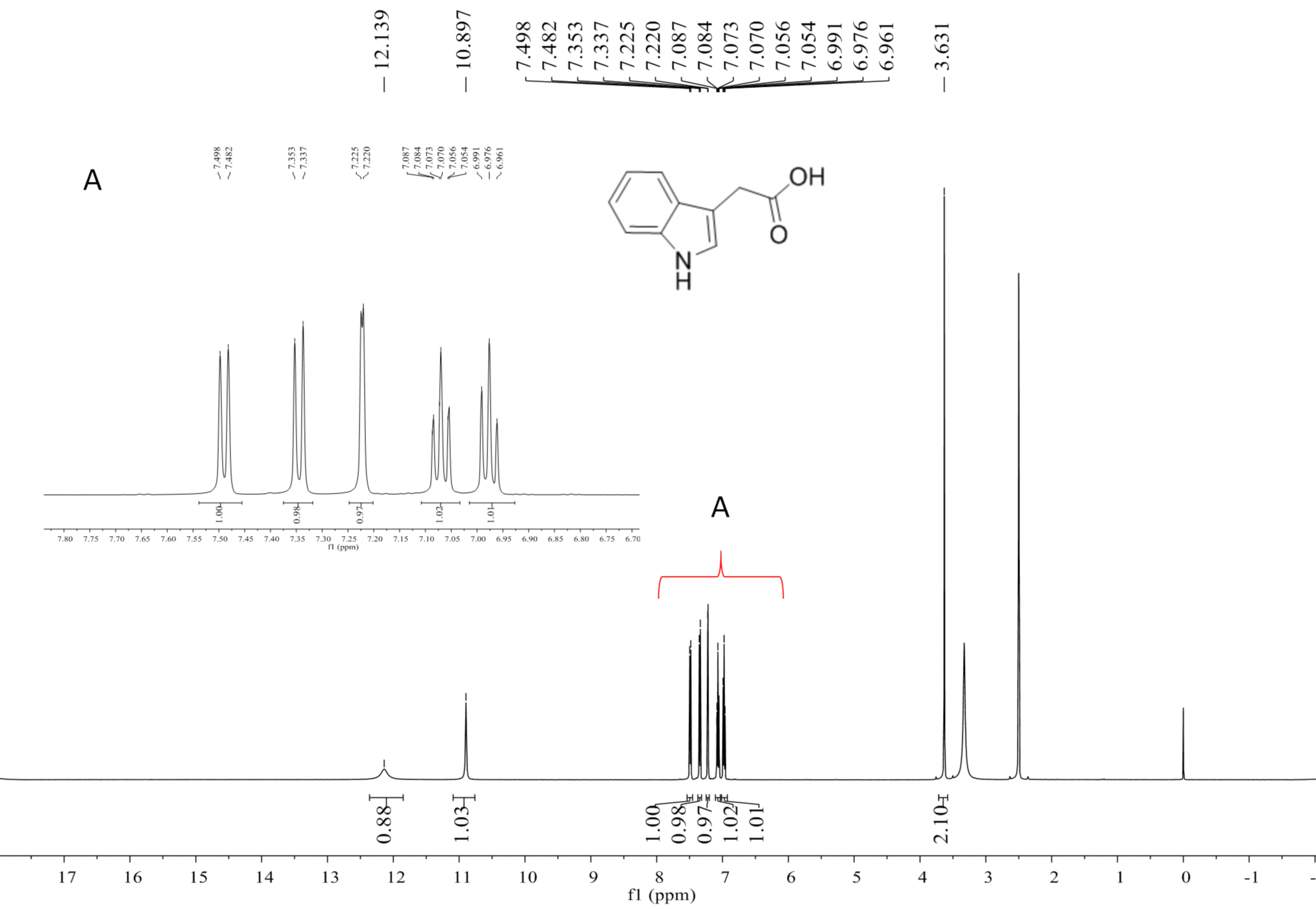

Supplement: Supplemental Information 4 — DMSO-d6, 500 MHz. [file peerj-08-9403-s004.png]

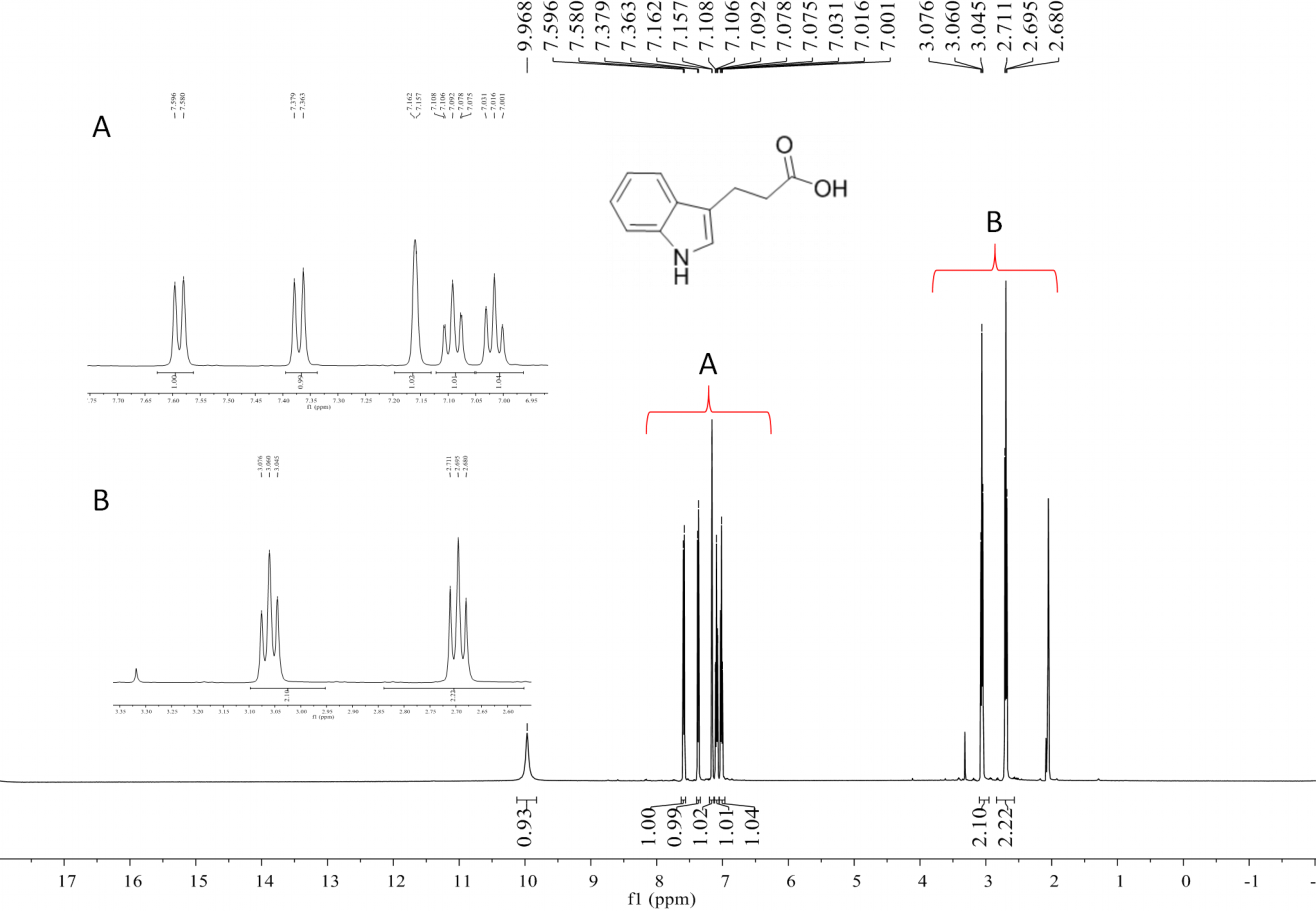

Supplement: Supplemental Information 5 — DMSO-d6, 500 MHz. [file peerj-08-9403-s005.png]

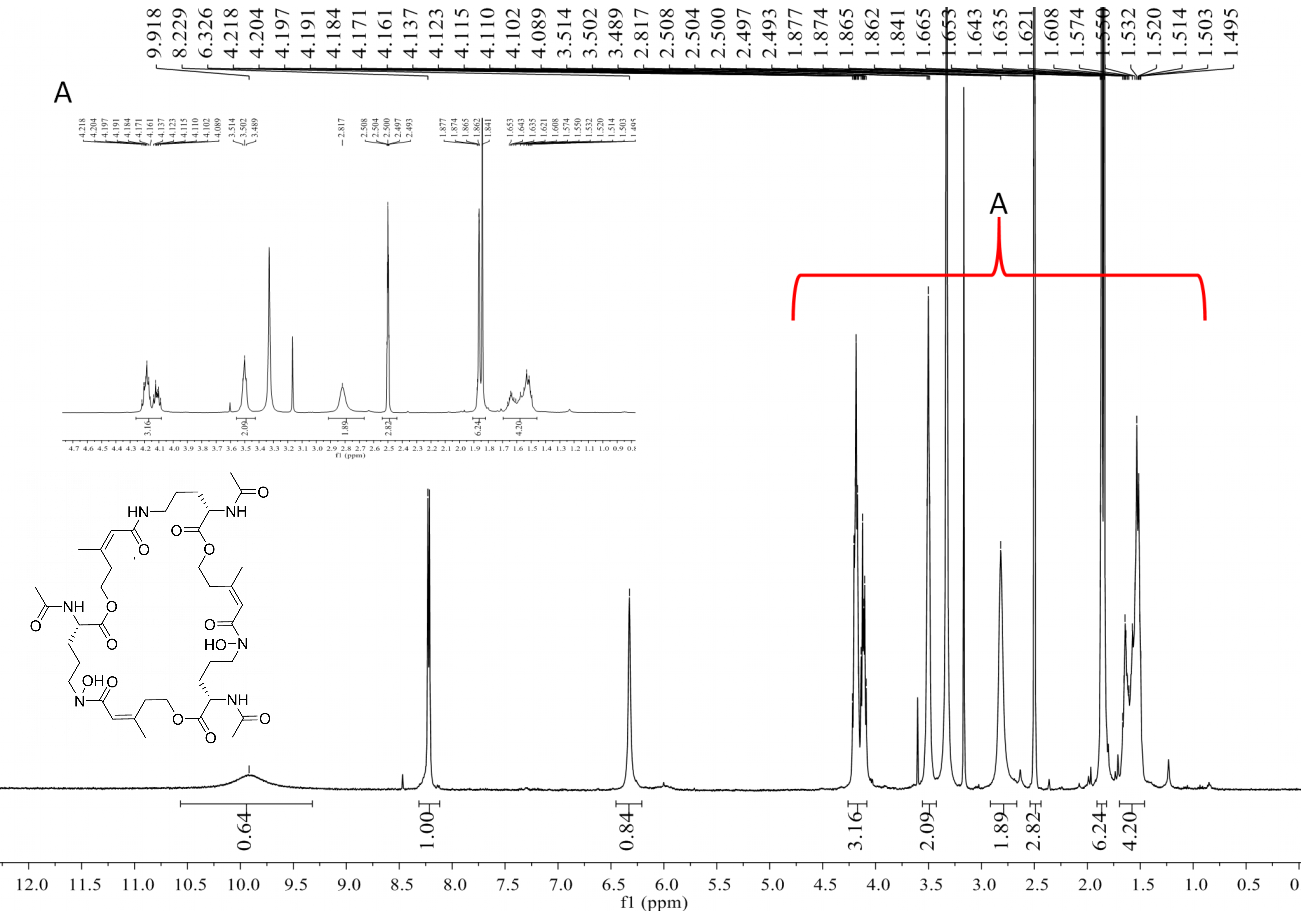

Supplement: Supplemental Information 6 — DMSO-d6, 500 MHz. [file peerj-08-9403-s006.png]

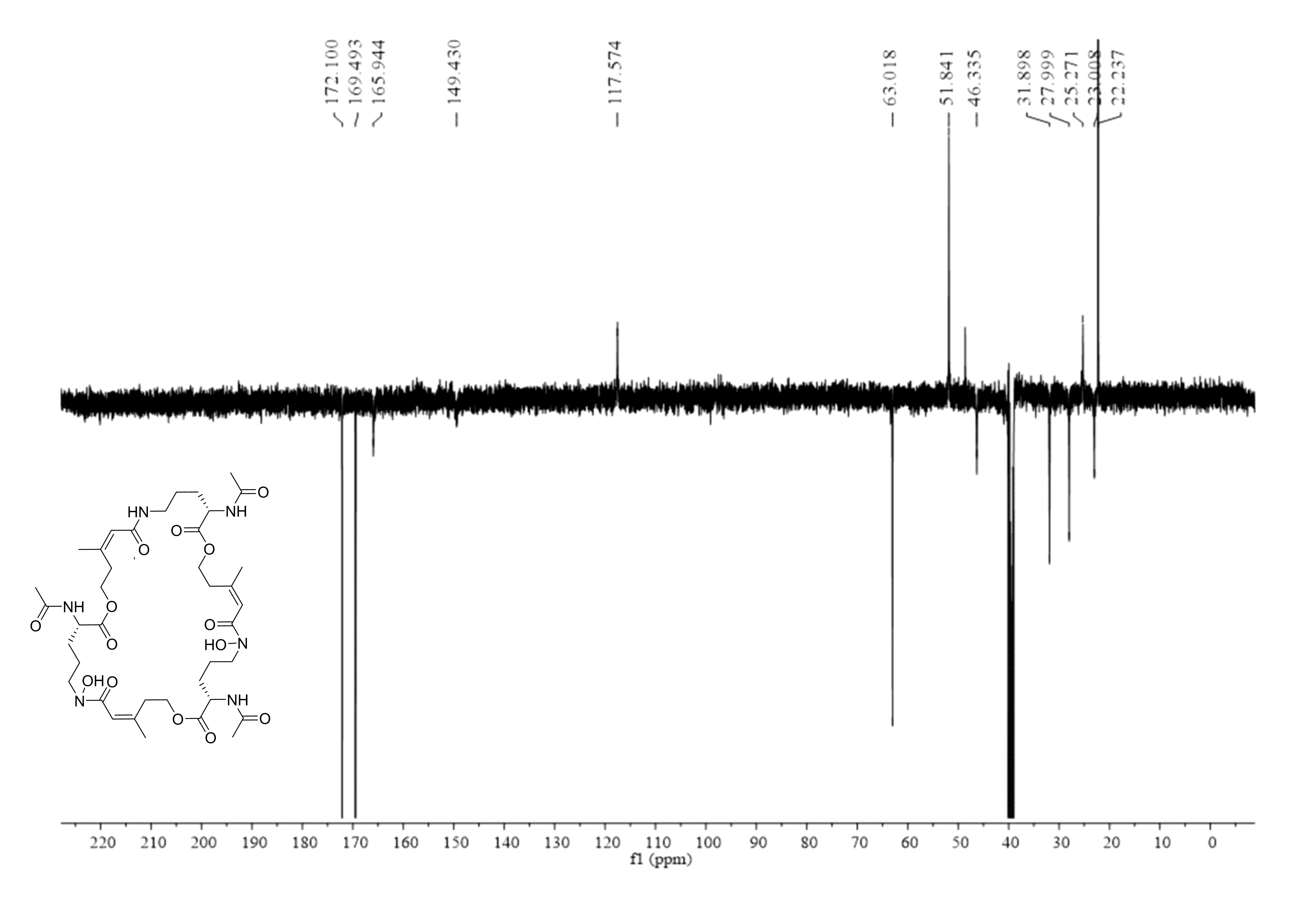

Supplement: Supplemental Information 7 — DMSO-d6, 125 MHz. [file peerj-08-9403-s007.png]

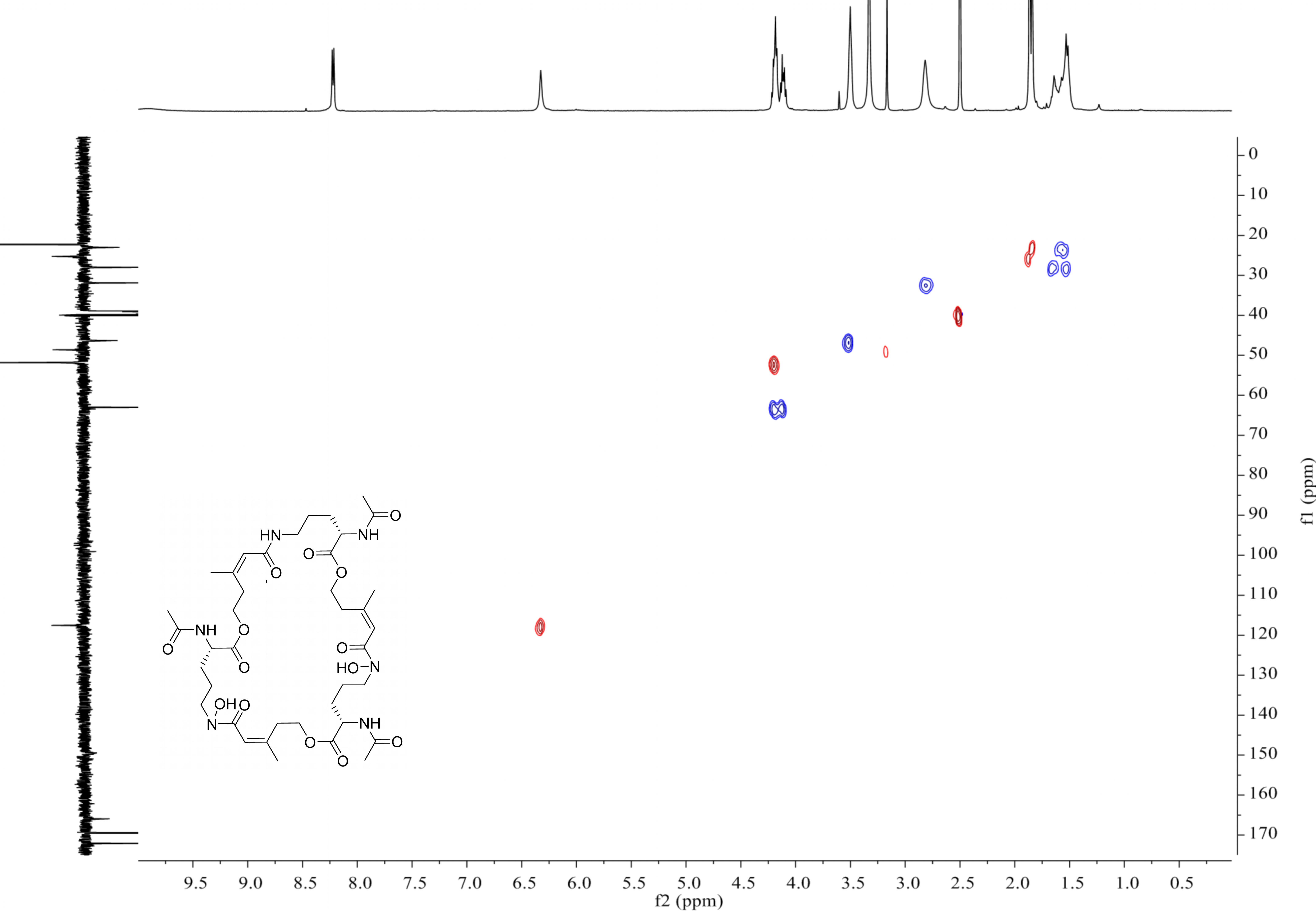

Supplement: Supplemental Information 8 — DMSO-d6, 145 MHz. [file peerj-08-9403-s008.png]

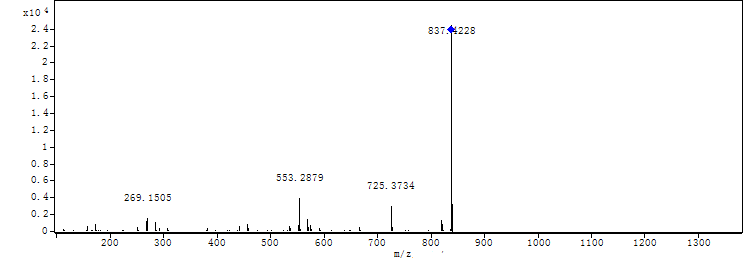

Supplement: Supplemental Information 9 — MS-MS spectrum was detected under 130.0 V. Compound 1 has three subunits, two of which have the same molecular weight (m/z 284.1366) and the other was different (m/z 268.1566). [file peerj-08-9403-s009.png]

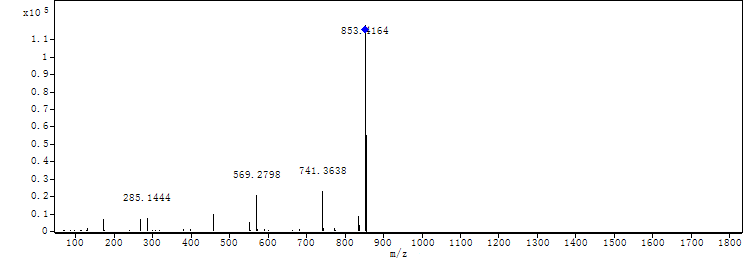

Supplement: Supplemental Information 10 — MS-MS spectrum of compound 2 was detected under 130.0 V. Compound 2 has three subunits of the same molecular mass (m/z 284.1366). [file peerj-08-9403-s010.png]

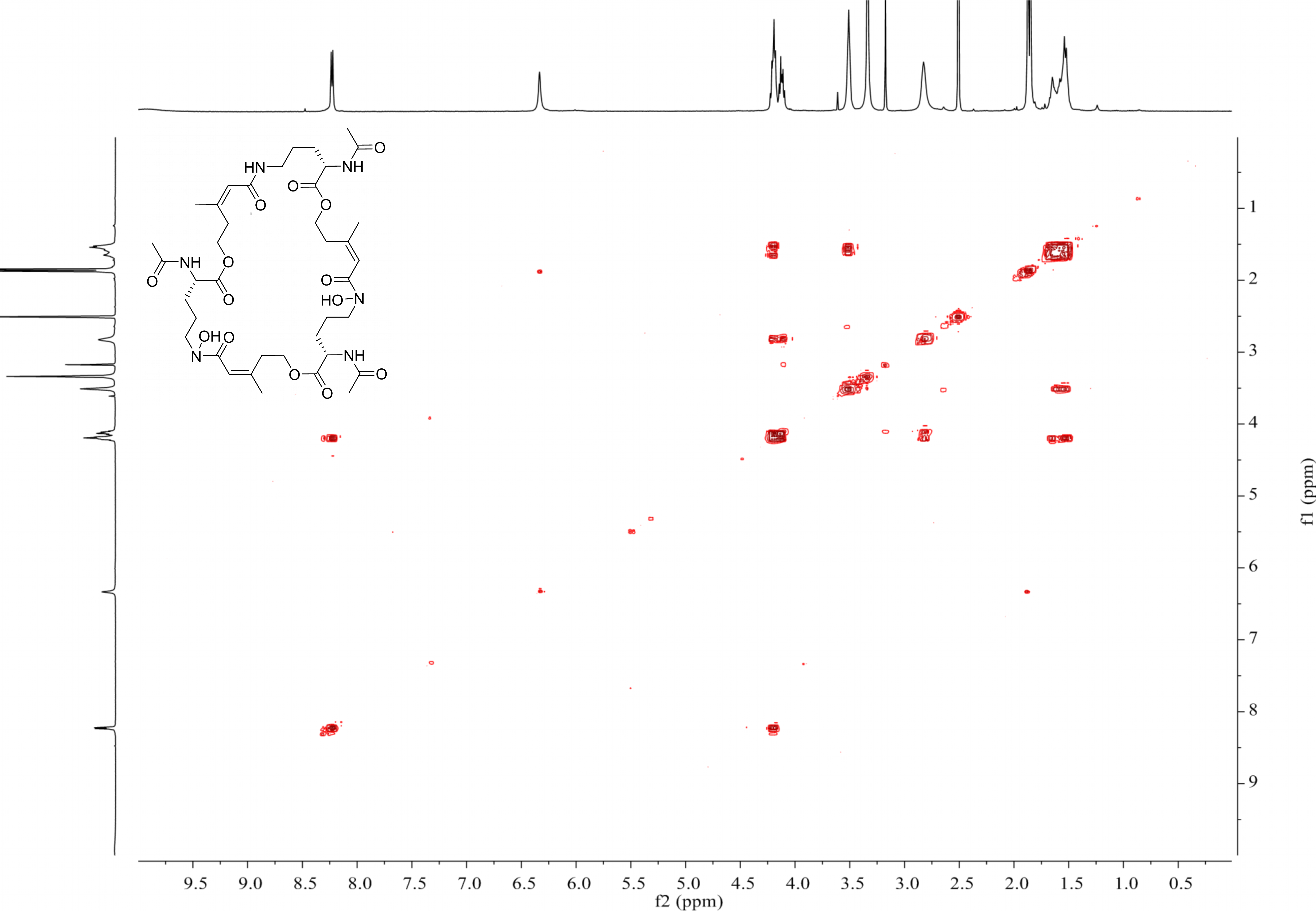

Supplement: Supplemental Information 11 — DMSO-d6, 8 MHz. [file peerj-08-9403-s011.png]

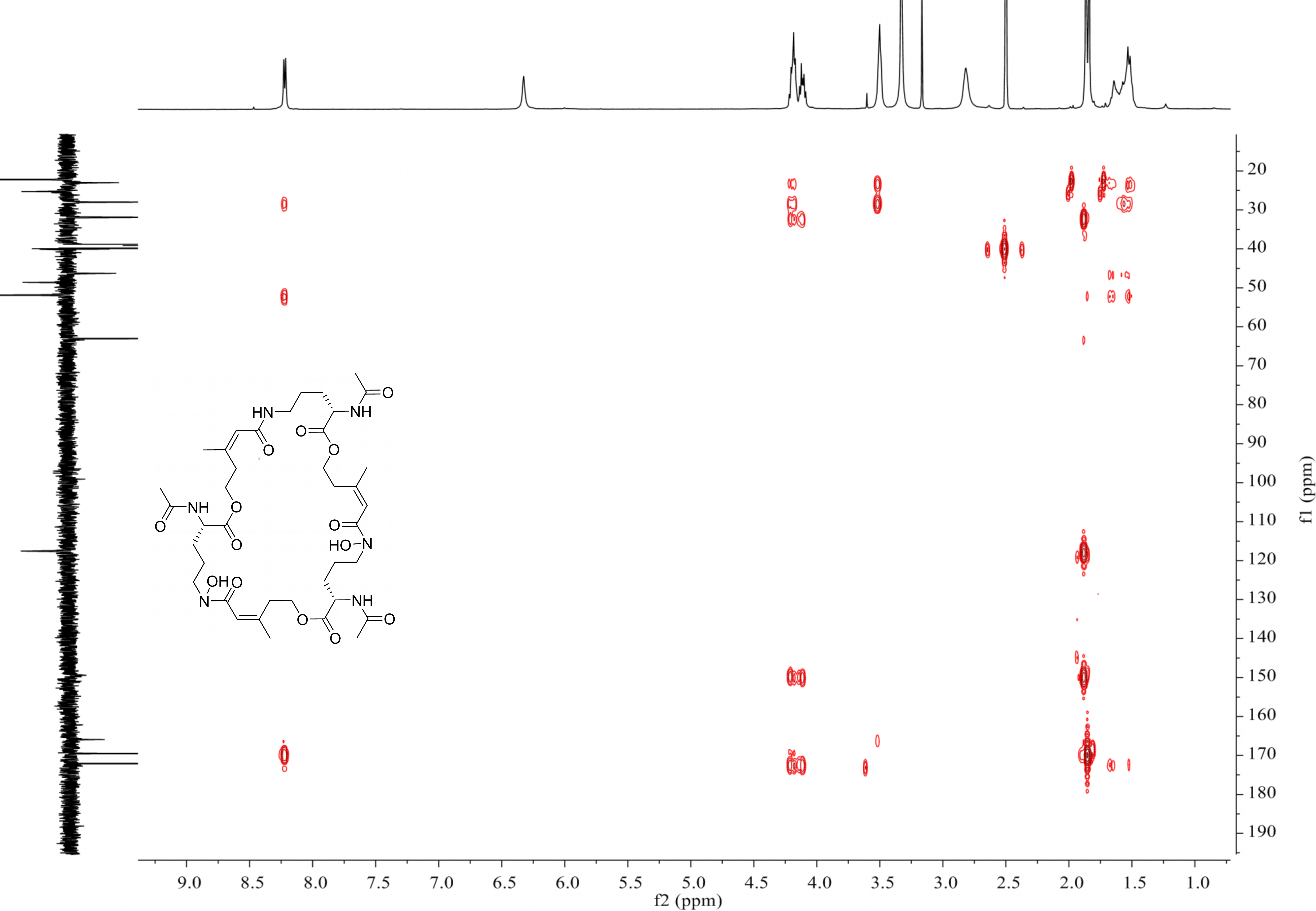

Supplement: Supplemental Information 12 — DMSO-d6, 8 MHz. [file peerj-08-9403-s012.png]

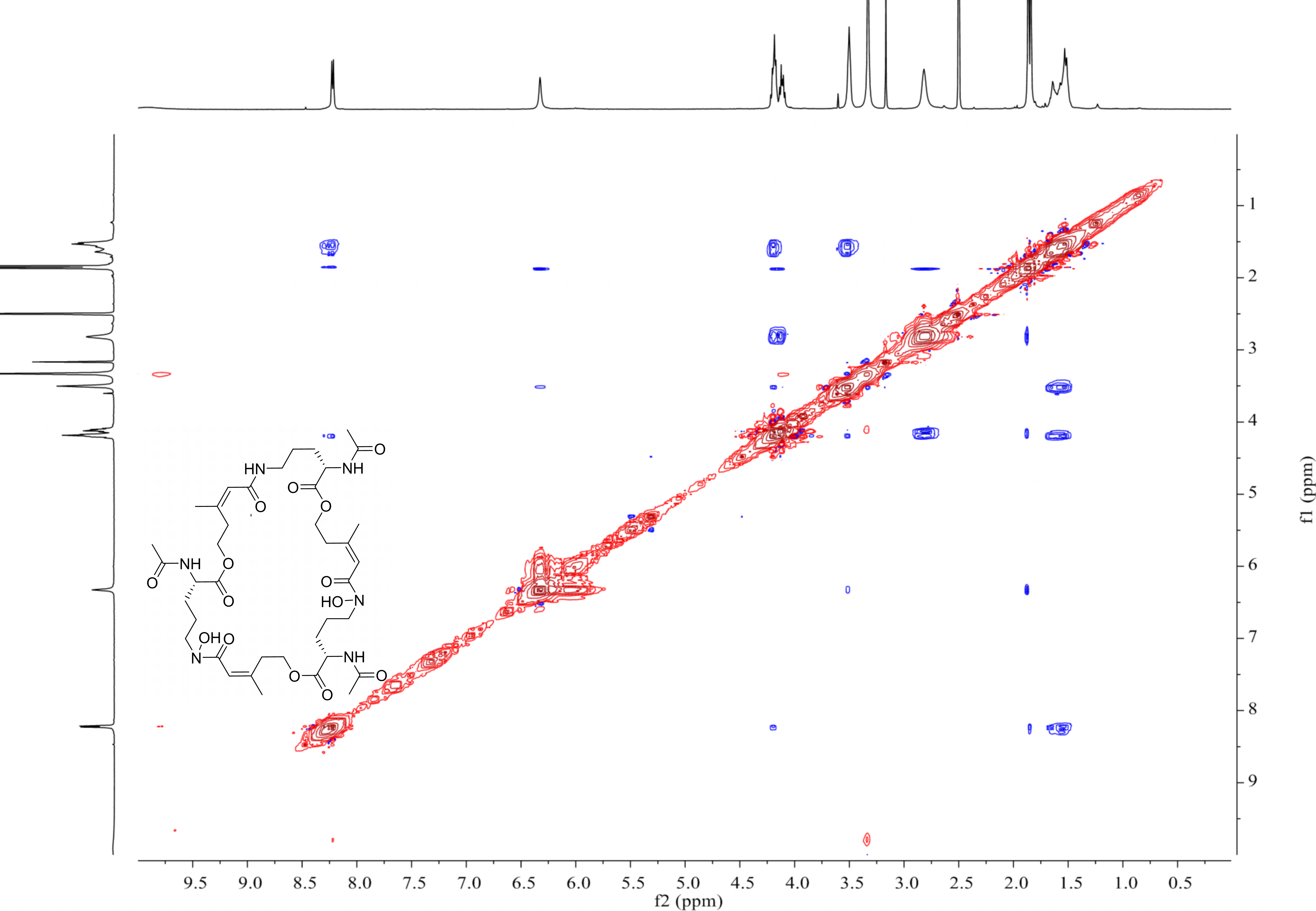

Supplement: Supplemental Information 13 — DMSO-d6. [file peerj-08-9403-s013.png]

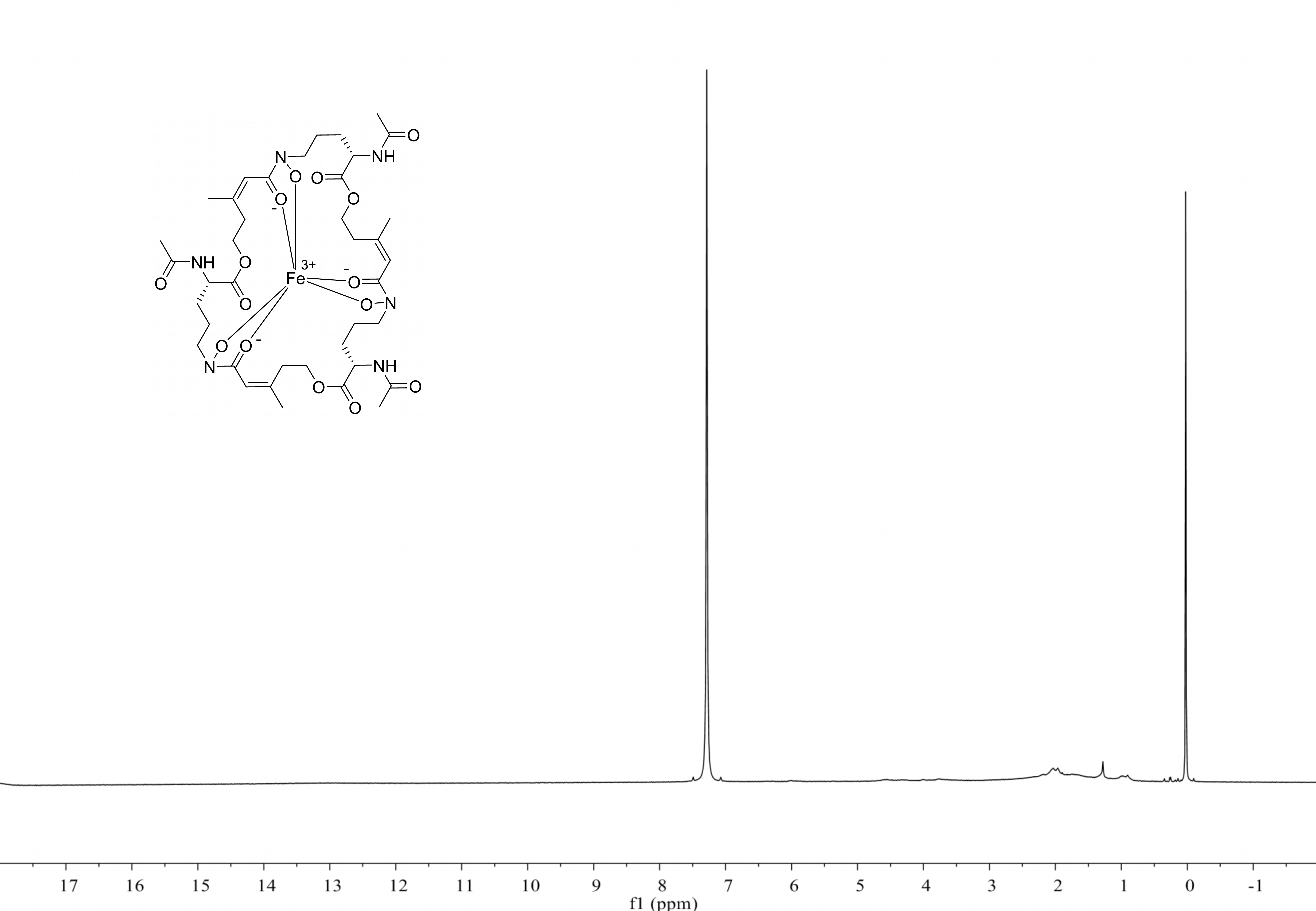

Supplement: Supplemental Information 14 — CDCl3, 500 MHz (no signal except solvent). [file peerj-08-9403-s014.png]

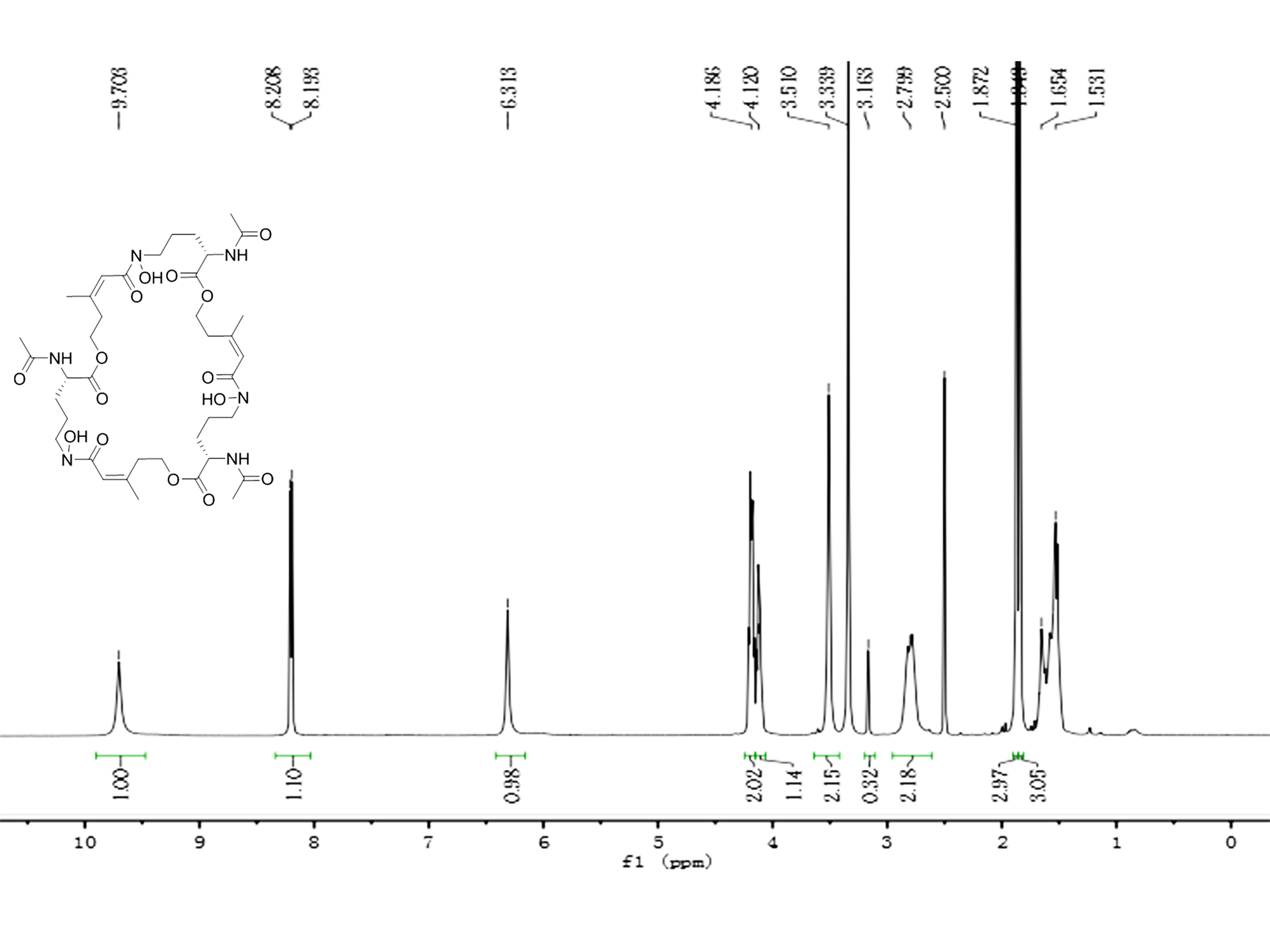

Supplement: Supplemental Information 15 — DMSO-d6, 500 MHz. [file peerj-08-9403-s015.png]

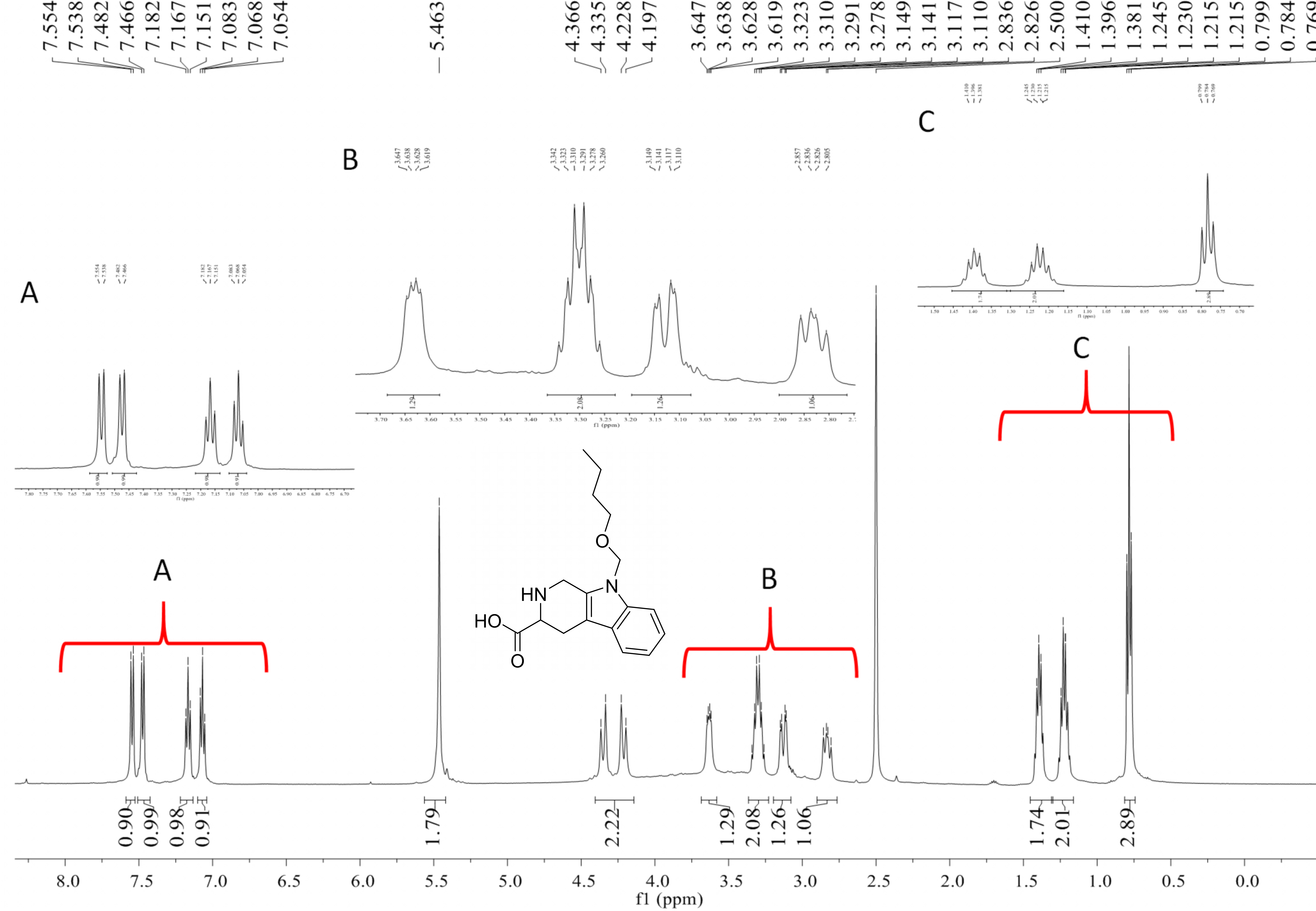

Supplement: Supplemental Information 16 — DMSO-d6, 500 MHz. [file peerj-08-9403-s016.png]

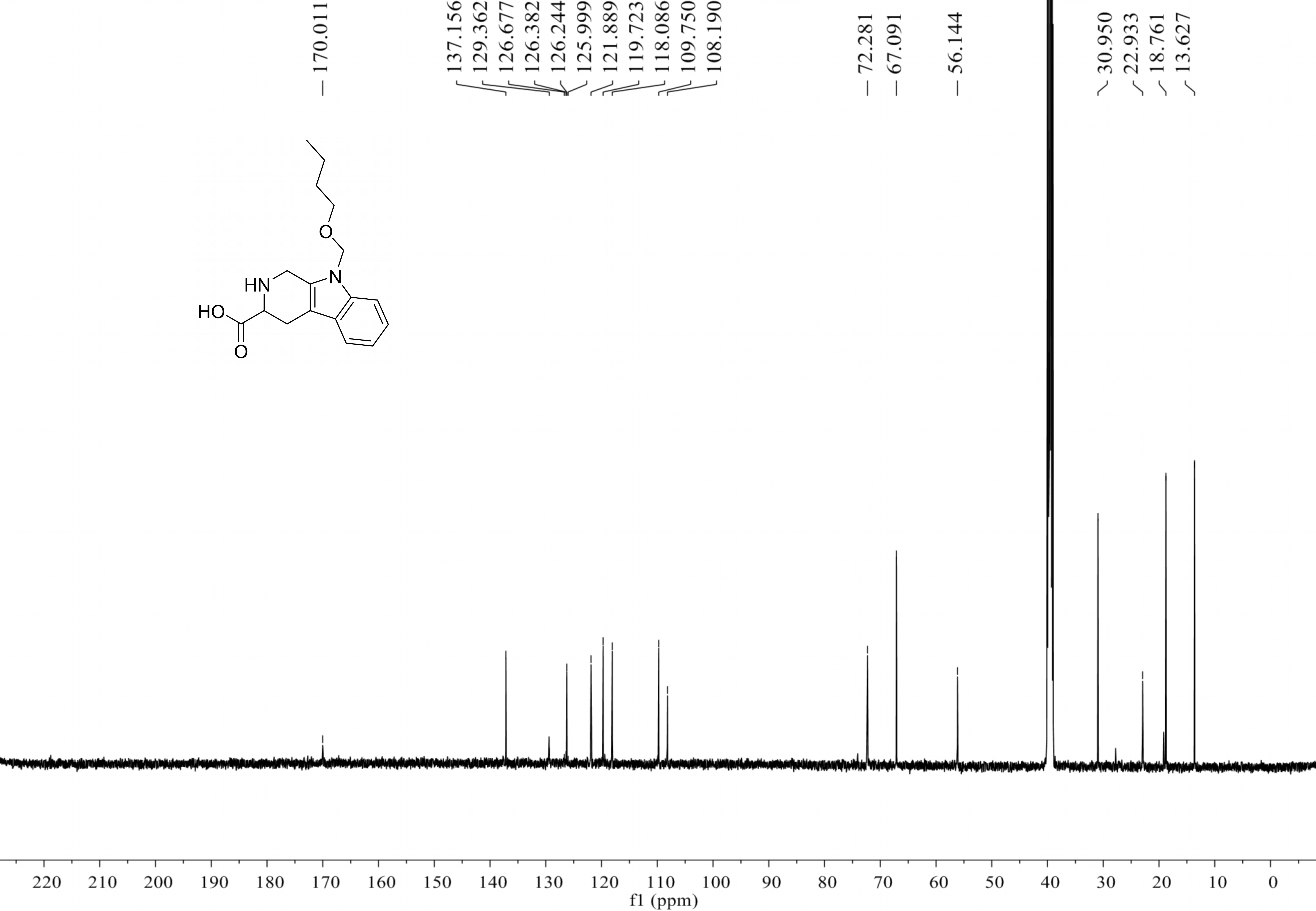

Supplement: Supplemental Information 17 — DMSO-d6, 125 MHz. [file peerj-08-9403-s017.png]

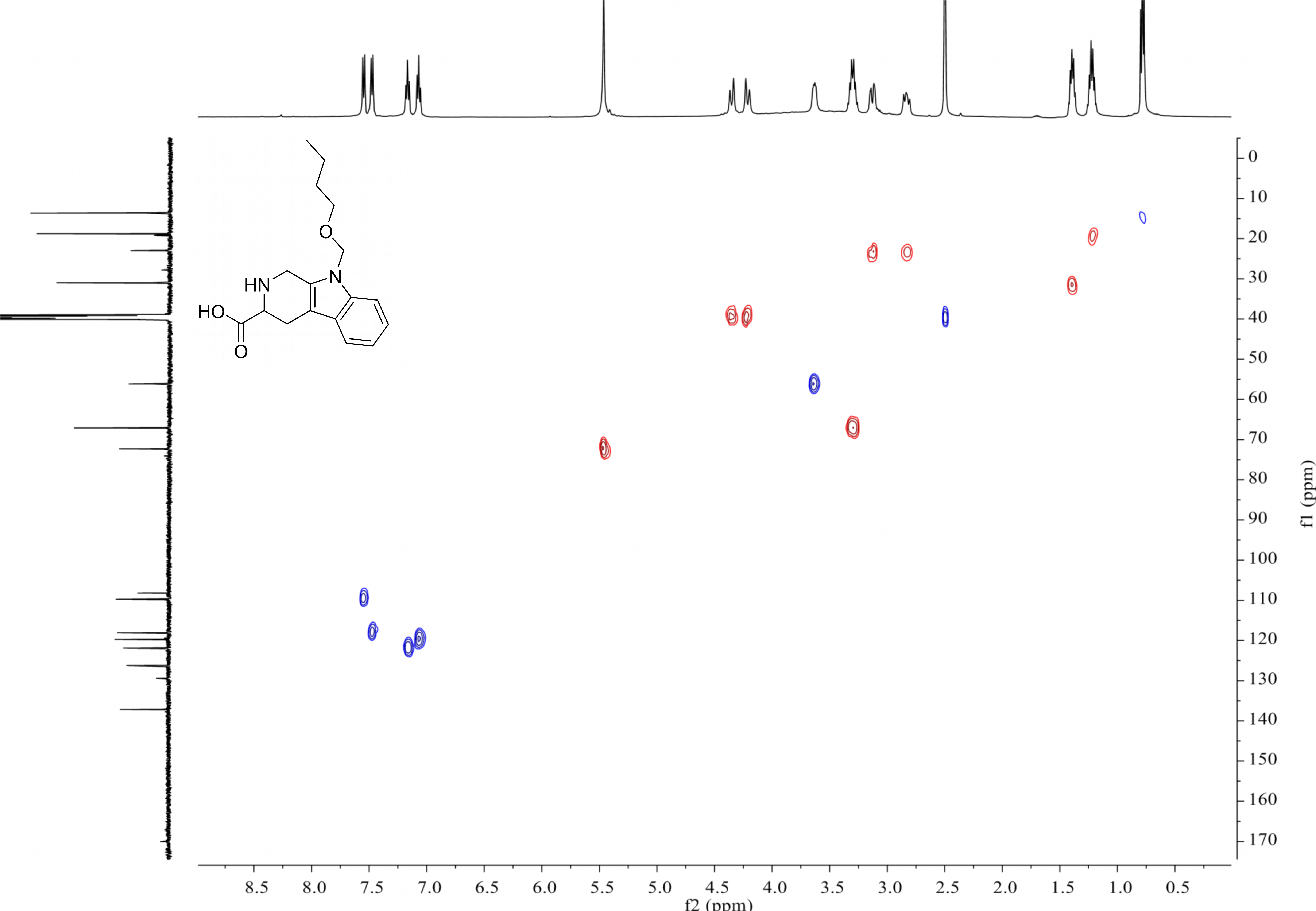

Supplement: Supplemental Information 18 — DMSO-d6, 145 MHz. [file peerj-08-9403-s018.png]

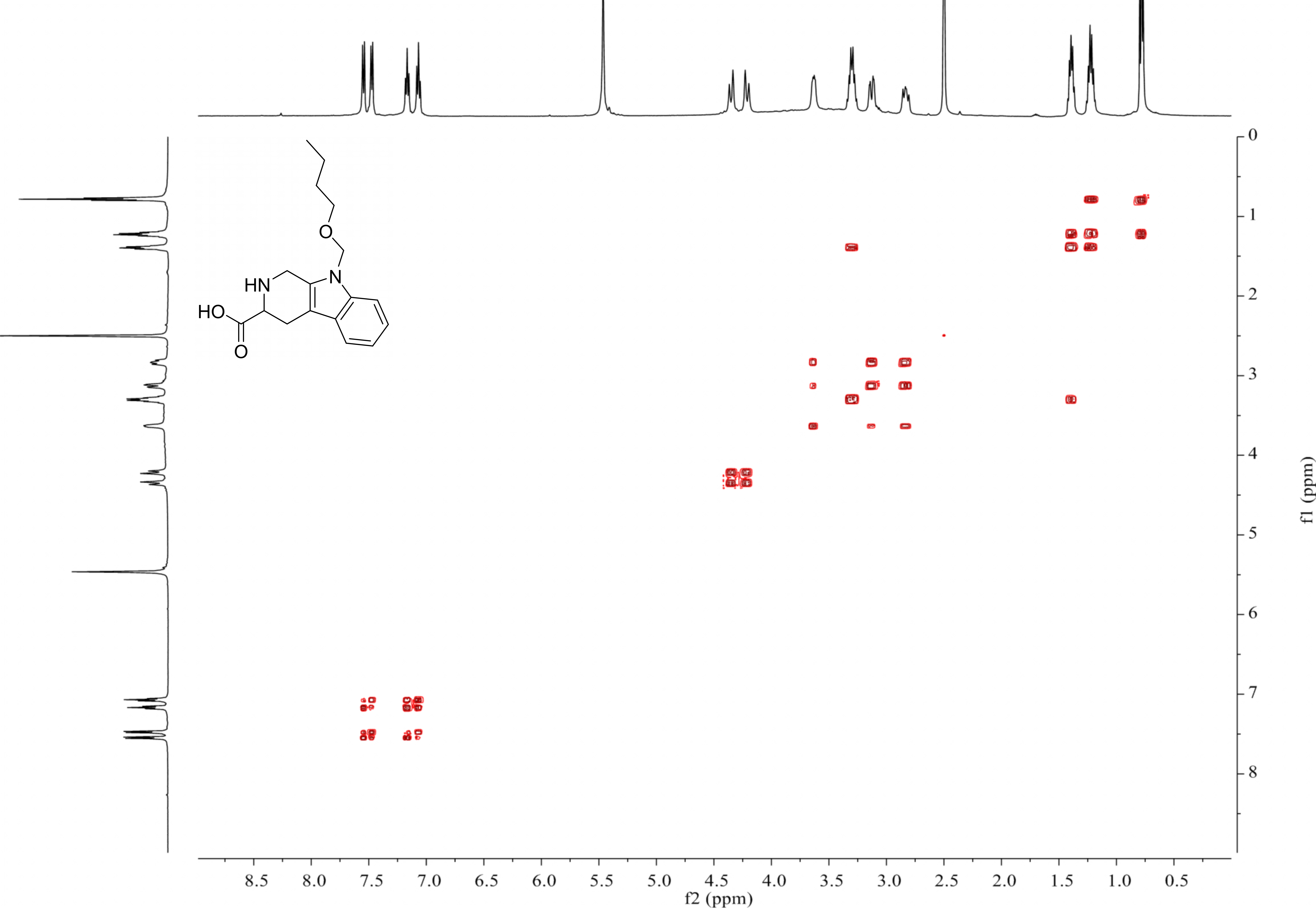

Supplement: Supplemental Information 19 — DMSO-d6, 8 MHz. [file peerj-08-9403-s019.png]

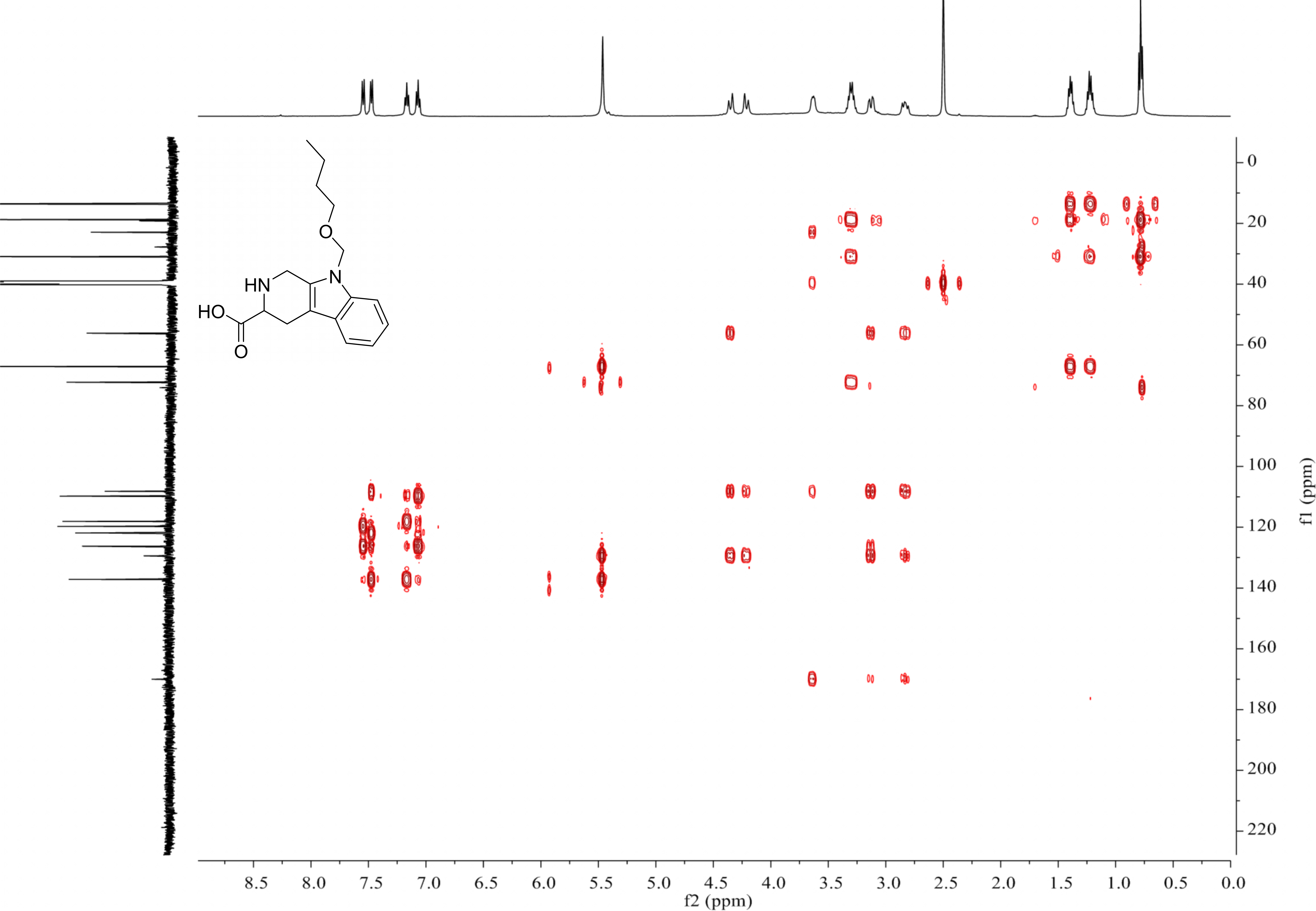

Supplement: Supplemental Information 20 — DMSO-d6, 8 MHz. [file peerj-08-9403-s020.png]

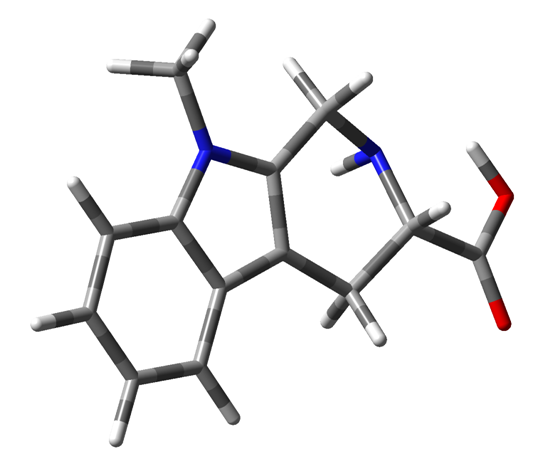

Supplement: Supplemental Information 21 — 4a-1 34.0743 kcal/mol, 95.1827%. [file peerj-08-9403-s021.png]

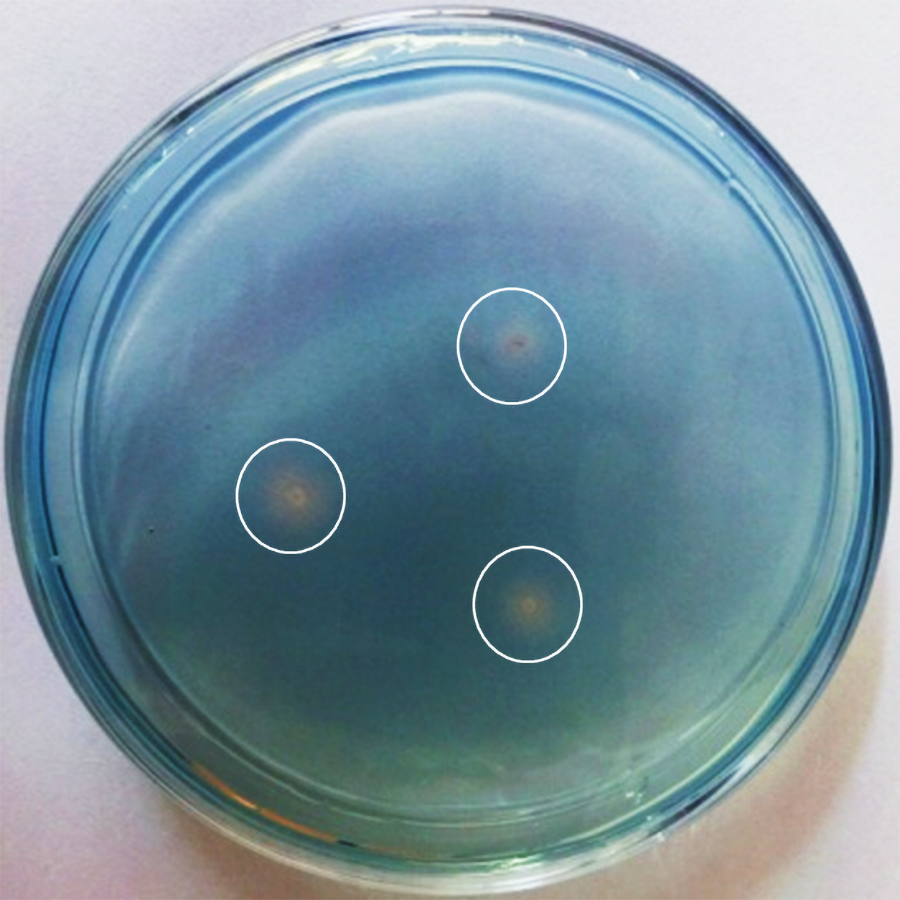

Supplement: Supplemental Information 22 — The CAS/HDTMA complexes were bound with ferric iron to produce a blue color as indicators. Siderophores could remove iron from the dye complex to turn it to light yellow. The light yellow ring around each colony appeared after seven days of growth indicating that P. triticisoli 1-18 had the ability of secreting siderophore and transferring iron from environment to the bacterial cells. [file peerj-08-9403-s022.png]
