# Supplementary material for: Siderophore and indolic acid production by Paenibacillus triticisoli BJ-18 and their plant growth-promoting and antimicrobe abilities
Source: PeerJ. 2020 Jul 14;8:e9403. doi: 10.7717/peerj.9403 (PMC7367057; doi:10.7717/peerj.9403)
Supplement: Supplemental Information 23 — The results of P. triticisoli BJ-18 were compared with the databases of mzCloud and Chemspider, indicating the existence of at least 101 compounds, including N_2-acetylornithine, which is the precursor of fusarinines. [file peerj-08-9403-s023.docx]

**Table S1.** Untargeted metabolomics profiling of *P. triticisoli* BJ-18

| Comp. | Formula | Molecular | RT | ChemSpider | mzCloud | mzVault |
| --- | --- | --- | --- | --- | --- | --- |
|  |  | Weight | [min] | Results | Results | Results |
| Carbohydrate and derivatives | | | | | | |
| Glyceraldehyde | C_3_H_6_O_3_ | 90.0316 | 1.293 | Full match | No results | No results |
| erythrose | C_4_H_8_O_4_ | 120.0421 | 1.289 | Full match | No results | No results |
| D-(-)-Fructose | C_6_H_12_O_6_ | 180.0633 | 1.221 | Not the top hit | Full match | No results |
| Sucrose | C_12_H_22_O_11_ | 342.11583 | 1.327 | Full match | Full match | Full match |
| Glucose 1-phosphate | C_6_H_13_O | 260.02928 | 1.18 | Not the top hit | Full match | No results |
| α-D-Glucose-1,6-bisphosphate | C_6_H_14_O_12_P_2_ | 339.99553 | 1.114 | Not the top hit | Full match | No results |
| trehalose 6-phosphate | C_12_H_23_O_14_P | 422.08226 | 1.18 | Full match | No results | No results |
| Amino acids and peptides | | | | | | |
| L-(+)-Alanine | C_3_H_7_NO_2_ | 89.04774 | 1.3 | Full match | No results | No results |
| L-(+)-Aspartic acid | C_4_H_7_NO_4_ | 133.03744 | 1.161 | Full match | No results | No results |
| L-(-)-Threonine | C_4_H_9_NO_3_ | 119.05808 | 1.303 | Full match | No results | No results |
| L-(+)-Lysine | C_6_H_14_N_2_O_2_ | 146.10544 | 1.343 | Full match | No results | No results |
| valeramide | C_5_H_11_NO | 101.08396 | 1.378 | Partial match | No results | Full match |
| D-(-)-Glutamine | C_5_H_10_N_2_O_3_ | 146.06901 | 1.248 | Full match | Full match | Full match |
| D-(+)-Proline | C_5_H_9_NO_2_ | 115.06307 | 8.79 | Full match | Full match | No results |
| D-(+)-Pyroglutamic Acid | C_5_H_7_NO_3_ | 129.04213 | 1.77 | Full match | No results | No results |
| L-Pyroglutamic acid | C_5_H_7_NO_3_ | 129.04213 | 1.418 | Full match | Full match | No results |
| 4-Oxoproline | C_5_H_7_NO_3_ | 129.04242 | 1.282 | Partial match | Full match | No results |
| Betaine | C_5_H_11_NO_2_ | 117.07862 | 1.324 | Partial match | Full match | No results |
| N-Acetyl-L-aspartic acid | C_6_H_9_NO_5_ | 175.0472 | 1.653 | Full match | No results | No results |
| D/L-Arginine | C_6_H_14_N_4_O_2_ | 174.11086 | 1.286 | Full match | Full match | No results |
| Prolylglycine | C_7_H_12_N_2_O_3_ | 172.08472 | 1.292 | Full match | No results | No results |
| N^2^-Acetylornithine | C_7_H_14_N_2_O_3_ | 174.10026 | 1.299 | Partial match | Full match | No results |
| L-Theanine | C_7_H_14_N_2_O_3_ | 174.0997 | 8.024 | Full match | No results | No results |
| Tyramine | C_8_H_11_NO | 137.08348 | 10.754 | Full match | No results | No results |
| Ne-Acetyllysine | C_8_H_16_N_2_O_3_ | 188.11589 | 1.295 | Full match | No results | No results |
| N^2^-succinyl-L-ornithine | C_9_H_16_N_2_O_5_ | 232.10571 | 1.236 | Full match | No results | No results |
| Capryloylglycine | C_10_H_19_NO_3_ | 201.1364 | 8.833 | No results | Full match | No results |
| Spermine | C_10_H_26_N_4_ | 202.2148 | 1.088 | Full match | No results | No results |
| L-Ala-Gamma-D-Glu-Meso-Diaminopimelic Acid | C_15_H_26_N_4_O_8_ | 390.175 | 1.168 | Full match | No results | No results |
| L-Saccharopine | C_11_H_20_N_2_O_6_ | 276.13191 | 1.181 | Full match | Full match | No results |
| (+/-)-pantetheine | C_11_H_22_N_2_O_4_S | 278.12997 | 7.213 | Full match | No results | No results |
| N-Acetylneuraminic acid | C_11_H_19_NO_9_ | 309.10585 | 1.266 | Full match | No results | No results |
| D-Sphingosine | C_18_H_37_NO_2_ | 299.28107 | 12.053 | No results | Full match | No results |
| Alcohols, aldehydes, ketones, fatty acids and lipids | | | | | | |
| Methylglyoxal | C_3_H_4_O_2_ | 72.02104 | 1.336 | Full match | No results | No results |
| D-(+)-Glyceric acid | C_3_H_6_O_4_ | 106.02643 | 1.22 | Full match | No results | No results |
| Succinic acid | C_4_H_6_O_4_ | 118.02653 | 1.232 | Full match | Full match | No results |
| 5-Aminovaleric acid | C_5_H_11_NO_2_ | 117.07883 | 1.379 | Partial match | Full match | No results |
| (+/-)-2-Hydroxyglutaric acid | C_5_H_8_O_5_ | 148.03706 | 1.219 | Full match | No results | No results |
| Aminolevulinic acid | C_5_H_9_NO_3_ | 131.05811 | 1.295 | Full match | No results | No results |
| 2,3-Dihydroxy-3-methyl-butanoic acid | C_5_H_10_O_4_ | 134.0578 | 1.29 | Full match | No results | No results |
| Hexanoic acid | C_6_H_12_O_2_ | 116.08368 | 7.445 | Full match | Full match | No results |
| Histidinol | C_6_H_11_N_3_O | 141.08962 | 1.063 | Full match | No results | No results |
| 4-Aminobenzoic acid | C_7_H_7_NO_2_ | 137.04714 | 1.356 | Full match | No results | No results |
| Caprylic acid | C_8_H_16_O_2_ | 144.11481 | 10.757 | Full match | Full match | No results |
| 8-Amino-7-oxononanoic acid | C_9_H_17_NO_3_ | 187.11997 | 9.62 | Full match | No results | No results |
| Decanoic acid | C_10_H_20_O_2_ | 172.14606 | 12.252 | Full match | Full match | No results |
| 4-Methoxycinnamaldehyde | C_10_H_10_O_2_ | 162.06734 | 13.257 | No results | Full match | No results |
| 3-Hydroxydecanoic acid | C_10_H_20_O_3_ | 188.14104 | 11.095 | No results | Full match | No results |
| Undecanoic acid | C_11_H_22_O_2_ | 186.16176 | 12.648 | No results | Full match | No results |
| Jasmonic acid | C_12_H_18_O_3_ | 210.12555 | 11.39 | No results | Full match | No results |
| 12-Hydroxydodecanoic acid | C_12_H_24_O_3_ | 216.17238 | 11.665 | No results | Full match | No results |
| Tridecylic acid | C_13_H_26_O_2_ | 214.19305 | 13.48 | No results | Full match | No results |
| 3,5-Tetradecadienoic acid | C1_4_H_24_O_2_ | 224.17655 | 12.628 | Full match | No results | No results |
| Pentadecanoic acid | C_15_H_30_O_2_ | 242.22409 | 14.056 | No results | Full match | No results |
| Palmitoleic acid | C_16_H_30_O_2_ | 254.2242 | 14.013 | Full match | Full match | No results |
| α-Humulene | C_15_H_24_ | 204.1868 | 13.828 | No results | No results | Full match |
| (-)-Caryophyllene oxide | C_15_H_24_O | 220.18171 | 13.451 | No results | Full match | No results |
| Tetradecanedioic acid | C_14_H_26_O_4_ | 258.18297 | 10.665 | No results | Full match | No results |
| 7Z-heptadecenoic acid | C_17_H_32_O_2_ | 268.23982 | 14.283 | Full match | No results | No results |
| Margaric acid | C_17_H_34_O_2_ | 270.25539 | 14.519 | Full match | No results | No results |
| 16-Hydroxyhexadecanoic acid | C_16_H_32_O_3_ | 272.23482 | 13.802 | No results | Full match | No results |
| Diosmetin | C_16_H_12_O_6_ | 300.06404 | 1.119 | No results | Full match | No results |
| Elaidolinolenic acid | C_18_H_30_O_2_ | 278.22421 | 13.933 | Full match | No results | No results |
| Oleic acid | C_18_H_34_O_2_ | 282.25552 | 14.483 | Full match | Full match | No results |
| Hexadecanedioic acid | C_16_H_30_O_4_ | 286.21419 | 11.779 | No results | Full match | No results |
| 9-hydroxy-10,12-octadecadienoic acid | C_18_H_32_O_3_ | 296.235 | 13.008 | No results | No results | Full match |
| 8Z,11Z,14Z-Eicosatrienoic acid | C_20_H_34_O_2_ | 306.25553 | 14.385 | No results | Full match | No results |
| Arachidic acid | C_20_H_40_O_2_ | 312.30256 | 14.882 | No results | No results | Full match |
| 20-Oxoleukotriene B4 | C_20_H_30_O_5_ | 350.20881 | 13.389 | Full match | No results | No results |
| Tetrahydrocurcumin | C_21_H_24_O_6_ | 372.15759 | 6.929 | Full match | No results | No results |
| Deoxycholic acid | C_24_H_40_O_4_ | 392.29234 | 13.047 | Full match | Full match | Full match |
| Cholic acid | C_24_H_40_O_5_ | 408.28724 | 12.308 | Partial match | Full match | Full match |
| Glycocholic acid | C_26_H_43_NO_6_ | 465.30833 | 12.038 | No results | Full match | Full match |
| Dipalmitoylphosphatidic acid | C_35_H_69_O_8_P | 648.4723 | 15.965 | Full match | No results | No results |
| Dilauroyl phosphatidylglycerol | C_30_H_59_O_10_P | 610.38399 | 14.888 | Full match | No results | No results |
| Nucleic acids | | | | | | |
| Guanine | C_5_H_5_N_5_O | 151.04869 | 1.347 | Full match | No results | No results |
| Xanthine | C_5_H_4_N_4_O_2_ | 152.03325 | 1.302 | Full match | Full match | No results |
| Dihydrothymine | C_5_H_8_N_2_O_2_ | 128.05801 | 1.336 | Full match | No results | No results |
| 2'-Deoxycytidine | C_9_H_13_N_3_O_4_ | 227.08967 | 1.31 | Full match | No results | No results |
| Uridine | C_9_H_12_N_2_O_6_ | 244.06923 | 1.3 | Full match | Full match | Full match |
| 5-aminoimidazole ribotide | C_8_H_14_N_3_O_7_P | 295.05718 | 1.262 | Full match | No results | No results |
| Vitamins | | | | | | |
| Nicotinic acid | C_6_H_5_NO_2_ | 123.0316 | 1.555 | Full match | Full match | Full match |
| Nicotinamide | C_6_H_6_N_2_O | 122.04761 | 1.454 | Partial match | Full match | Full match |
| (+)-Dethiobiotin | C_10_H_18_N_2_O_3_ | 214.13164 | 1.324 | Full match | No results | No results |
| Biotin | C_10_H_16_N_2_O_3_S | 244.08702 | 7.932 | Full match | No results | No results |
| Biotin l-Sulfoxide | C_10_H_16_N_2_O_4_S | 260.08179 | 6.665 | Full match | No results | No results |
| Flavin mononucleotide | C_17_H_21_N_4_O_9_P | 456.10451 | 7.032 | Full match | Full match | No results |
| Riboflavin | C_17_H_20_N_4_O_6_ | 376.138 | 7.927 | Full match | Full match | Full match |
| Choline | C_5_H_13_NO | 103.09966 | 1.282 | No results | Full match | No results |
| Cyclics | | | | | | |
| 8-Hydroxyquinoline | C_9_H_7_NO | 145.05213 | 12.814 | Full match | Full match | Full match |
| 2-hydroxyquinoline | C_9_H_7_NO | 145.05264 | 8.945 | Partial match | No results | Full match |
| Indole | C_8_H_7_N | 117.05731 | 6.349 | Full match | Full match | No results |
| Skatole | C_9_H_9_N | 131.07288 | 6.356 | No results | Full match | No results |
| 6-Methylquinoline | C_10_H_9_N | 143.07289 | 7.359 | No results | Full match | No results |
| lumichrome | C_12_H_10_N_4_O_2_ | 242.07921 | 9.882 | No results | No results | Full match |
| Indirubin | C_16_H_10_N_2_O_2_ | 262.07317 | 11.351 | No results | Full match | No results |
| Coproporphyrin III | C_36_H_38_N_4_O_8_ | 654.26604 | 12.468 | Full match | No results | No results |
| Alkaloids | | | | | | |
| Trigonelline | C_7_H_7_NO_2_ | 137.04708 | 14.241 | Partial match | Full match | No results |
| Acetylcarnitine | C_9_H_17_NO_4_ | 203.11563 | 6.98 | Full match | No results | No results |
| Oxymatrine | C_15_H_24_N_2_O_2_ | 264.1825 | 11.473 | No results | Full match | No results |
| Luotonin A | C_18_H_11_N_3_O | 285.08891 | 12.303 | No results | Full match | No results |
